# Risk adjustment models for interhospital comparison of CS rates using Robson’s ten group classification system and other socio-demographic and clinical variables

**DOI:** 10.1186/1471-2393-12-54

**Published:** 2012-06-21

**Authors:** Paola Colais, Maria P Fantini, Danilo Fusco, Elisa Carretta, Elisa Stivanello, Jacopo Lenzi, Giulia Pieri, Carlo A Perucci

**Affiliations:** 1 Department of Epidemiology, Regional Health Service, Lazio Region, Italy; 2 Department of Medicine and Public Health, University of Bologna, Bologna, Italy; 3 National Agency of Regional Health Services, Italy

## Abstract

**Background:**

Caesarean section (CS) rate is a quality of health care indicator frequently used at national and international level. The aim of this study was to assess whether adjustment for Robson’s Ten Group Classification System (TGCS), and clinical and socio-demographic variables of the mother and the fetus is necessary for inter-hospital comparisons of CS rates.

**Methods:**

The study population includes 64,423 deliveries in Emilia-Romagna between January 1, 2003 and December 31, 2004, classified according to theTGCS. Poisson regression was used to estimate crude and adjusted hospital relative risks of CS compared to a reference category. Analyses were carried out in the overall population and separately according to the Robson groups (groups I, II, III, IV and V–X combined). Adjusted relative risks (RR) of CS were estimated using two risk-adjustment models; the first (M1) including the TGCS group as the only adjustment factor; the second (M2) including in addition demographic and clinical confounders identified using a stepwise selection procedure. Percentage variations between crude and adjusted RRs by hospital were calculated to evaluate the confounding effect of covariates.

**Results:**

The percentage variations from crude to adjusted RR proved to be similar in M1 and M2 model. However, stratified analyses by Robson’s classification groups showed that residual confounding for clinical and demographic variables was present in groups I (nulliparous, single, cephalic, ≥37 weeks, spontaneous labour) and III (multiparous, excluding previous CS, single, cephalic, ≥37 weeks, spontaneous labour) and IV (multiparous, excluding previous CS, single, cephalic, ≥37 weeks, induced or CS before labour) and to a minor extent in groups II (nulliparous, single, cephalic, ≥37 weeks, induced or CS before labour) and IV (multiparous, excluding previous CS, single, cephalic, ≥37 weeks, induced or CS before labour).

**Conclusions:**

The TGCS classification is useful for inter-hospital comparison of CS section rates, but residual confounding is present in the TGCS strata.

## Background

Caesarean section (CS) rate is one of the most frequently used indicators of health care quality at the national and international level for clinical governance and outcome research. Hospitals and health-care systems are often compared on the basis of this indicator with the implicit assumption that lower rates reflect more appropriate practice, although the rate that defines optimum quality of care is undefined and seems to depend on the characteristics of the populations under study [1]. The World Health Organization has indicated that a CS rate greater than 10–15% is not justified in any region of the world. Rates are higher in developed countries, Latin America and the Caribbean, and lower in other developing countries [2-6].

In 2005, the Italian CS rate was the highest in Europe (38.5% vs. an average European rate of 23.7%) and one of the highest in the world [6]. In Italy, national CS rates have increased from 32% in 2001 to 38.5% in 2005. This increase was found both for primary CS and repeated CS. Primary caesarean deliveries contribute 2/3 to the overall CS rate, although the contribution of repeated CS is higher in regions with high overall CS rates [7]. Primary caesarean deliveries are an important target for reduction, because they lead to an increased risk for repeated caesarean delivery [8-10]. Therefore, some authors suggested to focus on primary CS for inter-hospital comparison and quality improvement [11], and others, based on evidence suggesting that non-vertex and multiple births may have better outcomes with cesarean deliveries [12], omitted these categories from the calculation of CS rates and focused on nulliparous term cephalic singleton (NTCS) deliveries.

In 2001, a new classification for CS known as the “ten group” (TGCS) or Robson classification was proposed. This classification system categorizes women into 10 mutually exclusive groups, considering the following *a priori* criteria: parity, the previous obstetric record of the woman, the course of labour, including pre-labour CS, and gestational age [11]. Several studies used the Robson classification system to compare CS rates within specific subsets of an obstetric population to overcome many of the historic controversies that have arisen when comparing overall caesarean rates among different populations [13-17]. A recent systematic review [18] supported the use of TGCS classification over other classifications based on characteristics of parturients for auditing purposes and comparison of CS rates across different settings.

The TGCS classifies CS according to the characteristics of each woman and her pregnancy. However, caesarean delivery has many other indications such as fetal distress, dystocia, placenta previa, HIV, and other conditions of the mother and foetus [19]. The failure to account for such patient-specific risk factors may lead to biased interhospital comparisons [20,21]. The possible confounding effect is caused by the heterogeneous distribution of CS risk factors across hospitals, that is not taken into account in the a priori Robson classification.

A recent study addressing interhospital comparison of CS rates in women with a primary CS and those with NTCS deliveries emphasized a differential need to adjust for clinical variables of the mother and the foetus and for socio-demographic characteristics of the mother [22]. Adjustment proved to be warranted when the indicator of interest was primary CS rate but not when the indicator was NTCS CS rate. The aim of the present study is to define a risk-adjustment model for interhospital comparison using TGCS classification and clinical and socio-demographic characteristics of the mother and the fetus not included in the TGCS classification that are indications for CS.

## Methods

### Study population

Deliveries in Emilia-Romagna Region (Italy) from January 1, 2003 through December 31, 2004 were extracted from Hospital Discharge Abstracts of mothers and their babies (SDO), and Birth Certificates (CedaP). Record linkage was performed between the SDO and CedaP databases.

The SDO data includes demographics (ID number, sex, date and place of birth, place of residence), discharge ID, admission and discharge dates, discharge diagnoses and procedures (International Classification of Diseases, 9^th^ Revision, Clinical Modification ICD-IX-CM), ward(s) of hospitalization, date(s) of in-hospital transfer, and the regional code of the admitting facility.

Birth Certificates include demographic data of the mother, information on presentation and multiple pregnancy (singleton cephalic, singleton breech, trasverse or oblique lie, etc.), parity (nulliparous, multiparous), the course of labour and delivery (spontaneous labour, induced labour or CS before labour) and gestational age (defined as the number of completed weeks at the time of birth).

To identify the delivery by Hospital Discharge Abstracts of mothers and their babies (SDO), we used three different sources of information: DRGs 370–375, procedure codes ICD-9-CM 72.*–74.* and diagnosis codes ICD-9-CM 640.*–676.*, V27.

DRG 370, procedure codes ICD-9-CM 74.0, 74.1, 74.2, 74.4, 74, 99 and diagnosis codes ICD-9-CM 669.7 were used to identify caesarean deliveries.

Mothers under the age of 11 or over the age of 50 years; mothers who were discharged from a hospital without an operating room; and infants with a birth weight under 550 g or over 6000 g were excluded.

CS rates were calculated as the ratio of caesarean deliveries to total deliveries.

Deliveries were retrospectively classified according to Robson’s Ten Group Classification System (TGCS) using information in the databases

The following socio-demographic variables were considered as potential risk factors for caesarean sections: maternal age (classified as <20, 20–34, or ≥35 years), citizenship (Italian, from developing countries, from developed countries other than Italy) and educational level (≤ 5, 6–8, 9–13, or ≥14 years). Maternal and neonatal clinical factors that constitute indication for CS were extracted using primary and secondary discharge diagnoses of Hospital Discharge Abstracts (see Additional file 1: Appendix A for the ICD-9-CM codes).

The study was carried out in compliance with the Italian law on privacy (Art. 20–21, DL 196/2003) and the regulations of the Regional Health Authority of Emilia-Romagna on data management. Access to the data was approved by the hospital trust administration. Data were anonymized at the regional statistical office where a unique identifier, the same for all databases, was assigned to each patient. This identifier does not allow to trace the patient’s identity and other sensitive data. When anonymized administrative data are used to inform health care planning activities, the study is exempt from notification to the Ethics Committee and no specific written consent is needed to use patient information stored in the hospital databases.

### Statistical analysis

Analyses were initially carried out on the entire population. We then analyzed the I–IV Robson groups separately, and the V–X Robson groups taken together. The last six Robson groups accounted for about 20% of all deliveries and could not be analyzed separately because of the small number of deliveries in these groups by hospital. Crude and adjusted relative risks of CS for each hospital were calculated using as the reference category hospitals with the lowest CS rates. These hospitals were identified by means of a recursive procedure developed by P.Re.Val.E. Project [23]. Adjusted RR of CS (caesarean section risk for patients admitted to a specific hospital vs. caesarean section risk for patients admitted to the reference category) were obtained by using modified Poisson regression models based on the Huber sandwich estimate, that improves efficiency in mean-variance relationship.

Specifically, two risk adjustment models were set up. The first model (M1) was built using TGCS as the only potential confounding factor. The second model (M2) included, in addition to TGCS, a number of potential confounders (demographic and clinical variables related to the mother and foetus) selected according to available scientific evidence. These include age, citizenship, severe co-morbid illness of the mother, diabetes, hypertension, HIV, lung diseases, ante-partum haemorrhage/abruption placentae/placenta praevia, eclampsia/pre-eclampsia, foetal-pelvic disproportion/excessive development of the infant, polyhydramnios, oligohydramnios, isoimmunisation, premature rupture of the membranes, abortion threats/assisted fecundation, congenital malformation, problem of the amnios, post-maturity and macrosomia, intrauterine growth retardation (see Additional file 1 for the ICD-9-CM codes). A stepwise selection procedure (significance level for entry of 0.10 and 0.05 for stay), was used to remove variables unrelated to CS.

Stratum-specific models were defined for groups I to IV and V-X that included only clinical and demographic variables selected using a stepwise selection procedure.

Adjusted relative risks (RRs) and percentage variations between crude and adjusted RRs by hospital were then calculated to evaluate the amount of confounding. The presence of confounding was defined as a percentage variation ≥10% between crude and adjusted RRs [24,25]. The same hospitals selected as reference group in the overall population were used as the reference group in the stratified analyses. The significance level for the RR was set at 5% (p < 0.05). All analyses were performed using SAS Version 8.02.

## Results

**Figure 1:**
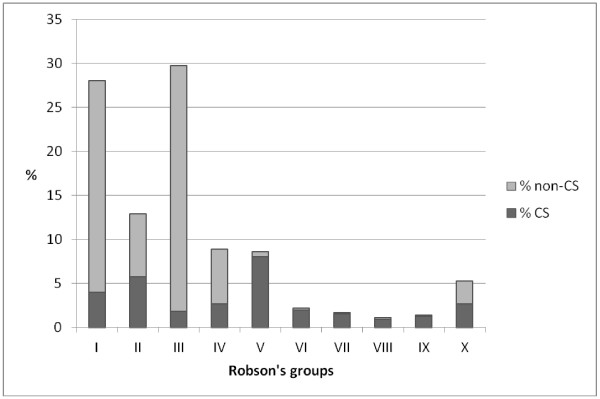
**Percentage distribution of deliveries by TGCS over the total number of deliveries.** Light gray: non-CS; gray: CS.

A total of 64,423 deliveries in Emilia-Romagna occurred between January 1, 2003, and December 31, 2004. The overall crude CS rate was 30.4%, and the CS rate in the reference group was 23.1%. Figure 1 shows the TGCS distribution in the study population and the proportion of CS in each group.

The first Robson group was the most frequent (28.0% of deliveries), while group VIII was the less frequent (1.1%). The first four groups constitute approximately the 80% of the entire population. Group V had the highest CS rate (93.6%), while group III showed the lowest recourse to CS, with a rate of 6.3%. Table 1 reports the number of caesarean deliveries, crude and adjusted caesarean section RRs, their statistical significance, and the percentage variation by hospital. The RR percentage variations by hospital estimated using the M1 model were very similar with those estimated using M2 model. The M1 adjusted RRs led to percentage variations greater than 10% in 16 out of 25 hospitals, while the M2 adjusted RRs led to percentage variations greater than 10% in 15 out of 25 hospitals.

**Table 1:** Crude and adjusted c-section RRs of hospitals

**Hospital**	**n**	**Crude RR**	**p–value**	Adj* RR	**p–value**	**% variation†**	**Adj‡ RR**	**p–value**	**% variation†**
A	2,377	1.39	<0.001	1.19	<0.001	−14.4	1.18	<0.001	−15.1
B	1,306	1.36	<0.001	1.25	<0.001	−8.1	1.26	<0.001	−7.4
C	935	1.05	0.428	1.00	0.908	−4.8	1.01	0.877	−3.8
D	344	1.27	0.006	1.35	<0.001	6.3	1.37	<0.001	7.9
E	1,468	1.34	<0.001	1.12	0.001	−16.4	1.14	<0.001	−14.9
F	1,677	1.41	<0.001	1.12	0.001	−20.6	1.09	0.006	−22.7
G	1,615	1.22	<0.001	1.08	0.026	−11.5	1.11	0.003	−9.0
H	459	1.71	<0.001	1.34	<0.001	−21.6	1.31	<0.001	−23.4
I	1,430	1.51	<0.001	1.26	<0.001	−16.6	1.21	<0.001	−19.9
J	2,096	1.39	<0.001	1.18	<0.001	−15.1	1.17	<0.001	−15.8
K	1,854	1.28	<0.001	1.25	<0.001	−2.3	1.24	<0.001	−3.1
L	4,389	1.02	0.550	0.90	<0.001	−11.8	0.90	<0.001	−11.8
M	603	1.82	<0.001	1.77	<0.001	−2.7	1.62	<0.001	−11.0
N	547	1.38	<0.001	1.13	0.044	−18.1	1.13	0.038	−18.1
O	1,923	1.02	0.616	1.09	0.019	6.9	1.05	0.188	2.9
P	2,120	1.38	<0.001	1.28	<0.001	−7.2	1.28	<0.001	−7.2
Q	4,377	1.14	<0.001	1.02	0.431	−10.5	1.03	0.261	−9.6
R	4,488	1.45	<0.001	1.29	<0.001	−11.0	1.29	<0.001	−11.0
S	598	2.73	<0.001	1.75	<0.001	−35.9	1.68	<0.001	−38.5
T	304	2.45	<0.001	1.76	<0.001	−28.2	1.72	<0.001	−29.8
U	4,591	1.58	<0.001	1.32	<0.001	−16.5	1.26	<0.001	−20.3
V	4,430	1.26	<0.001	1.06	0.009	−15.9	1.03	0.159	−18.3
W	5,635	1.34	<0.001	1.13	<0.001	−15.7	1.11	<0.001	−17.2
X	6,374	1.44	<0.001	1.35	<0.001	−6.2	1.31	<0.001	−9.0
Y	1,770	1.33	<0.001	1.25	<0.001	−6.0	1.23	<0.001	−7.5

* Adjusted for Robson class.

† The % variation is computed as (crude RR-adj RR)*100/crude RR.

‡ Adjusted for: age, citizenship, severe co-morbid illness of the mother, HIV, diabetes, hypertension, lung diseases, ante-partum haemorrhage/abruption placentae/placenta praevia, eclampsia/pre-eclampsia, foetal-pelvic disproportion/excessive development of the infant, foetal anomalies, RH-isoimmunisation, polyhydramnios, oligohydramnios, premature rupture of the membranes, problem of the amnios, congenital malformation, intrauterine growth retardation, post-maturity and macrosomia, Robson group.

Tables 2, 3, 4, 5 provide the number of caesarean deliveries, crude and adjusted caesarean section RRs, their statistical significance, and the percentage variation by hospital in groups I, II, III, IV. In group I (Table 2), 10 hospitals had a percentage variation greater than 10%, with a very high reduction in adjusted compared to the crude RR for hospital M (36.9%). In group II no percentage variation ≥10% was observed. In group III (Table 4), 12 hospitals had a percentage variation in relative risk higher than 10 %, and again hospital M proved to have the largest value (36.6%). In group IV 7 hospitals exhibited variations ≥10 % (Table 5). Lastly, Table 6 reports the number of caesarean deliveries, crude and adjusted caesarean section RRs, their statistical significance, and the percentage variation by hospital for Robson groups V–X. We found that only 2 hospitals (L, T) had a percentage variation greater than 10%.

**Table 2:** Crude and adjusted c-section RRs of hospitals in group I

**Hospital**	**n**	**Crude RR**	**p–value**	Adj* RR	**p–value**	**% variation†**
A	707	0.94	0.638	1.08	0.555	14.9
B	397	0.99	0.953	1.12	0.467	13.1
C	268	0.42	0.003	0.47	0.008	11.9
D	107	0.70	0.303	0.74	0.386	5.7
E	448	1.48	0.002	1.53	<0.001	3.4
F	416	1.80	<0.001	1.77	<0.001	−1.7
G	508	0.92	0.586	1.02	0.887	10.9
H	112	1.17	0.543	1.26	0.333	7.7
I	305	1.60	0.001	1.73	<0.001	8.1
J	488	1.21	0.161	1.32	0.037	9.1
K	572	1.38	0.008	1.49	0.001	8.0
L	1,151	0.73	0.01	0.76	0.017	4.1
M	243	3.85	<0.001	2.43	<0.001	−36.9
N	143	2.55	<0.001	1.96	<0.001	−23.1
O	582	1.35	0.013	1.31	0.016	−3.0
P	616	1.44	0.001	1.51	<0.001	4.9
Q	1,177	0.30	<0.001	0.34	<0.001	13.3
R	1,239	1.12	0.272	1.22	0.043	8.9
S	99	3.31	<0.001	2.62	<0.001	−20.8
T	69	3.66	<0.001	2.57	<0.001	−29.8
U	1,382	1.95	<0.001	1.84	<0.001	−5.6
V	1,333	0.80	0.047	0.83	0.095	3.7
W	1,284	1.19	0.081	1.30	0.007	9.2
X	1,965	2.47	<0.001	2.21	<0.001	−10.5
Y	564	1.33	0.021	1.28	0.036	−3.8

* Adjusted for: age, citizenship, severe co-morbid illness of the mother, HIV, diabetes, hypertension, ante-partum haemorrhage/abruption placentae/placenta praevia, eclampsia/pre-eclampsia, foetal-pelvic disproportion/excessive development of the infant, foetal anomalies, RH-isoimmunisation, polyhydramnios, oligohydramnios, abortion threats/assisted fecundation, congenital malformation, intrauterine growth retardation, post-maturity and macrosomia.

† The % variation is computed as (crude RR-adj RR)*100/crude RR.

**Table 3:** Crude and adjusted c-section RRs of hospitals in group II

**Hospital**	**n**	**Crude RR**	**p–value**	Adj* RR	**p–value**	**% variation†**
A	269	1.61	<0.001	1.52	<0.001	−5.6
B	145	1.54	<0.001	1.51	<0.001	−1.9
C	99	1.04	0.756	0.99	0.952	−4.8
D	37	2.13	<0.001	2.15	<0.001	0.9
E	260	1.11	0.257	1.13	0.159	1.8
F	347	1.24	0.006	1.25	0.004	0.8
G	324	1.18	0.040	1.22	0.012	3.4
H	81	1.78	<0.001	1.63	<0.001	−8.4
I	202	1.36	<0.001	1.35	<0.001	−0.7
J	315	1.09	0.330	1.09	0.297	0.0
K	226	1.53	<0.001	1.46	<0.001	−4.6
L	504	0.99	0.871	0.98	0.787	−1.0
M	54	0.65	0.087	0.64	0.070	−1.5
N	118	0.78	0.112	0.79	0.130	1.3
O	143	0.86	0.244	0.85	0.228	−1.2
P	369	1.41	<0.001	1.39	<0.001	−1.4
Q	644	1.22	0.003	1.20	0.007	−1.6
R	630	1.34	<0.001	1.33	<0.001	−0.7
S	113	2.26	<0.001	2.14	<0.001	−5.3
T	36	2.11	<0.001	2.05	<0.001	−2.8
U	405	1.62	<0.001	1.54	<0.001	−4.9
V	674	1.08	0.267	1.05	0.482	−2.8
W	779	1.22	0.002	1.21	0.002	−0.8
X	649	0.72	<0.001	0.75	<0.001	4.2
Y	266	1.51	<0.001	1.47	<0.001	−2.6

* Adjusted for: age, severe co-morbid illness of the mother, diabetes, ante-partum haemorrhage/abruption placentae/placenta praevia, eclampsia/pre-eclampsia, foetal-pelvic disproportion/excessive development of the infant, premature rupture of the membranes, post-maturity and macrosomia.

† The % variation is computed as (crude RR-adj RR)*100/crude RR.

**Table 4:** Crude and adjusted c-section RRs of hospitals in group III

**Hospital**	**n**	**Crude RR**	**p–value**	Adj* RR	**p–value**	**% variation†**
A	684	0.94	0.770	1.04	0.855	10.6
B	408	1.13	0.614	1.27	0.295	12.4
C	327	0.35	0.021	0.38	0.034	8.6
D	120	1.15	0.733	1.18	0.669	2.6
E	347	0.86	0.604	0.92	0.755	7.0
F	379	0.24	0.005	0.21	0.002	−12.5
G	393	0.76	0.342	0.89	0.671	17.1
H	120	0.96	0.924	1.14	0.771	18.8
I	460	0.90	0.671	1.00	0.999	11.1
J	616	1.60	0.007	1.68	0.001	5.0
K	586	1.57	0.012	1.70	0.002	8.3
L	1,295	0.73	0.077	0.77	0.127	5.5
M	150	5.82	<0.001	3.69	<0.001	−36.6
N	106	5.21	<0.001	5.04	<0.001	−3.3
O	717	1.70	0.001	1.72	<0.001	1.2
P	576	1.72	0.002	1.76	0.001	2.3
Q	1,325	0.31	<0.001	0.35	<0.001	12.9
R	1,282	1.42	0.015	1.45	0.009	2.1
S	103	2.68	0.001	2.96	<0.001	10.4
T	77	5.08	<0.001	3.50	<0.001	−31.1
U	1,283	2.54	<0.001	2.21	<0.001	−13.0
V	1,182	0.58	0.008	0.54	0.003	−6.9
W	1,664	1.20	0.189	1.25	0.101	4.2
X	1,926	3.40	<0.001	2.93	<0.001	−13.8
Y	491	1.17	0.466	1.12	0.579	−4.3

* Adjusted for: age, severe co-morbid illness of the mother, HIV, diabetes, hypertension, lung diseases, ante-partum haemorrhage/abruption placentae/placenta praevia, eclampsia/pre-eclampsia, foetal-pelvic disproportion/excessive development of the infant, foetal anomalies, RH-isoimmunisation, polyhydramnios, oligohydramnios, problem of the amnios, congenital malformation, intrauterine growth retardation, post-maturity and macrosomia.

† The % variation is computed as (crude RR-adj RR)*100/crude RR.

**Table 5:** Crude and adjusted c-section RRs of hospitals in group IV

**Hospital**	**n**	**Crude RR**	**p–value**	Adj* RR	**p–value**	**% variation†**
A	188	1.42	0.004	1.23	0.070	−13.4
B	105	1.80	<0.001	1.58	<0.001	−12.2
C	73	1.51	0.013	1.47	0.017	−2.6
D	35	2.36	<0.001	2.50	<0.001	5.9
E	127	1.08	0.621	1.07	0.637	−0.9
F	187	0.82	0.213	0.85	0.289	3.7
G	120	1.02	0.930	1.04	0.819	2.0
H	44	1.70	0.005	1.52	0.020	−10.6
I	168	1.47	0.002	1.41	0.004	−4.1
J	242	1.30	0.025	1.16	0.178	−10.8
K	162	1.50	0.001	1.41	0.003	−6.0
L	316	0.95	0.647	0.92	0.487	−3.2
M	43	0.64	0.206	0.62	0.157	−3.1
N	91	0.26	0.001	0.27	0.001	3.8
O	143	0.58	0.010	0.57	0.009	−1.7
P	225	1.22	0.102	1.13	0.287	−7.4
Q	398	1.28	0.015	1.20	0.059	−6.3
R	410	1.76	<0.001	1.69	<0.001	−4.0
S	71	2.49	<0.001	2.30	<0.001	−7.6
T	40	2.26	<0.001	1.91	<0.001	−15.5
U	370	1.77	<0.001	1.56	<0.001	−11.9
V	266	0.96	0.755	0.87	0.250	−9.4
W	671	1.17	0.102	1.18	0.074	0.9
X	527	0.47	<0.001	0.48	<0.001	2.1
Y	151	1.17	0.274	1.04	0.777	−11.1

* Adjusted for: age, severe co-morbid illness of the mother, lung diseases, ante-partum haemorrhage/abruption placentae/placenta praevia, eclampsia/pre-eclampsia, foetal-pelvic disproportion/excessive development of the infant, oligohydramnios, premature rupture of the membranes, abortion threats/assisted fecundation, congenital malformation, post-maturity and macrosomia.

† The % variation is computed as (crude RR-adj RR)*100/crude RR.

**Table 6:** Crude and adjusted c-section RRs of hospitals in groups V–X

**Hospital**	**n**	**Crude RR**	**p–value**	Adj* RR	**p–value**	**% variation†**	**Adj‡ RR**	**p–value**	**% variation†**
A	529	1.11	<0.001	1.09	<0.001	−1.8	1.11	<0.001	0.0
B	251	1.17	<0.001	1.14	<0.001	−2.6	1.13	<0.001	−3.4
C	168	1.14	<0.001	1.12	<0.001	−1.8	1.12	<0.001	−1.8
D	45	1.10	0.202	1.07	0.271	−2.7	1.02	0.729	−7.3
E	286	1.07	0.057	1.06	0.046	−0.9	1.06	0.027	−0.9
F	348	1.01	0.760	1.03	0.285	2	1.00	0.984	−1.0
G	270	1.10	0.006	1.11	<0.001	0.9	1.13	<0.001	2.7
H	102	1.18	<0.001	1.15	<0.001	−2.5	1.15	<0.001	−2.5
I	295	1.20	<0.001	1.15	<0.001	−4.2	1.09	<0.001	−9.2
J	435	1.11	<0.001	1.13	<0.001	1.8	1.09	<0.001	−1.8
K	308	1.02	0.650	1.05	0.145	2.9	1.05	0.074	2.9
L	1,123	0.77	<0.001	0.90	<0.001	16.9	0.91	<0.001	18.2
M	113	1.13	0.004	1.19	<0.001	5.3	1.12	<0.001	−0.9
N	89	1.06	0.277	1.05	0.311	−0.9	1.00	0.954	−5.7
O	338	0.99	0.850	1.08	0.014	9.1	1.04	0.143	5.1
P	334	1.09	0.004	1.15	<0.001	5.5	1.15	<0.001	5.5
Q	833	1.08	0.002	1.11	<0.001	2.8	1.11	<0.001	2.8
R	927	1.12	<0.001	1.20	<0.001	7.1	1.23	<0.001	9.8
S	212	1.21	<0.001	1.30	<0.001	7.4	1.28	<0.001	5.8
T	82	1.25	<0.001	1.16	<0.001	−7.2	1.12	0.001	−10.4
U	1,151	0.97	0.234	0.99	0.626	2.1	1.00	0.810	3.1
V	975	1.11	<0.001	1.14	<0.001	2.7	1.09	<0.001	−1.8
W	1,237	1.02	0.447	1.05	0.015	2.9	1.03	0.157	1.0
X	1,307	1.11	<0.001	1.19	<0.001	7.2	1.14	<0.001	2.7
Y	298	1.10	0.004	1.15	<0.001	4.5	1.13	<0.001	2.7

* Adjusted for Robson group.

† The % variation is computed as (crude RR-adj RR)*100/crude RR.

‡ Adjusted for: age, citizenship, severe co-morbid illness of the mother, HIV, diabetes, hypertension, ante-partum haemorrhage/abruption placentae/placenta praevia, eclampsia/pre-eclampsia, foetal-pelvic disproportion/excessive development of the infant, foetal anomalies, polyhydramnios, oligohydramnios, premature rupture of the membranes, abortion threats/assisted fecundation, congenital malformation, intrauterine growth retardation, post-maturity and macrosomia, multiple pregnancy, previous CS, cephalic/breech presentation.

## Discussion

The aim of the present study was to define a risk-adjustment model for inter-hospital comparison, using TGCS classification and variables not included in the TGCS classification that are indications for CS. Our results indicate that TGCS classification should be used to control for the hospital case mix in terms of parity, presentation, gestational age and multiple pregnancy. However, some residual variability, in the overall population and in the I and III groups, is accounted for by clinical and socio-demographic confounders.

Several studies used different risk adjustment [26-28] techniques to compare CS rates across hospitals. The stratified analyses proposed by Robson with “a priori” criteria of classification seem to overcome the problem of risk adjustment. However, our results suggest that the TGCS classification is not sufficient to remove case mix differences present in the first four TGCS groups, especially groups I and III. Brennan et al. [29], recently demonstrated a wide variation of caesarean section rates in women in spontaneous cephalic term labour (I and III TGCS groups) among 9 international “third level” hospitals and suggested the need to verify the role of other potential confounding factors when comparative evaluation is carried out. Our results incorporate in the analyses some clinical and socio-demographic CS risk factors related to mother and foetus, not included in the TGCS, but do not consider the organizational and process variables such as midwifery care, use of oxytocin to correct dystocia, intrapartum foetal blood sampling mentioned by Brennan et al. [29].

One of the strength of the present study is the opportunity to use two current administrative databases with a very good record linkage (higher then 95%) and to take advantage of data collected from two different sources. In this study, caesarean section occurrences were evaluated using discharge record data. Accuracy, completeness, and quality of records may differ from hospital to hospital, however the CS rates and proportion of all patients in Robson’s groups (data not shown) are similar to those reported in other studies [30,31]. The potential for inconsistencies in coding discharge records may challenge the accuracy of the assessment of outcome and of risk factors. Missing information on important risk factors and errors in coding may in fact lead to subsequent errors in adjustment and this represent a limit of the study. Finally, part of the limitations of administrative data may be due to the basic tension which exists between using the same data for reimbursement and for measuring quality. “When the use is reimbursement, there is a tendency to perform coding quickly and to maximize the coding of complications and co-morbidities. When the use is to assess quality, however, it is important for coders to have a complete record and to restrict diagnosis coding to conditions that affect patient care [32].” For instance, hypertension and diabetes may intervene in the algorithm used to determine the case mix of an admission and thus be rewarding in financial terms, whereas this may not be the case for labour induction and history of a previous caesarean. Nevertheless, administrative databases are widely available at the national and regional level, and are currently utilized to compare outcomes, including CS, of inpatient care in Italy [33]. Risk adjustment models should be time and population specific, and have recently proved to be useful for monitoring caesarean section rates and for interhospital comparison [26]. Methods used to develop models based on administrative information can potentially be generalized to other populations.

The TGCS is a good tool for clinical audit practices as it enables professionals to compare their CS practice with homogeneous a priori risk populations. Since low-risk deliveries both in nulliparous and multiparous women are an important target for reduction because in these categories the large majority of inappropriate CS can be found, our analytical method may be useful to partial out the effect of clinical and demographic variables in the TGCS groups. Furthermore, it is important to focus on the first four TGCS groups that in our country represent, given the low fertility rate (1.28 for years 2000–2005) [33] and current obstetric practice in relation to the management of deliveries, about two thirds of all deliveries [34] because in these groups it is most likely to find inappropriate CS.

Reducing the number of unnecessary CS in low-risk women is also a good strategy to indirectly reduce the CS in women with previous CS.

In conclusion, our results indicate that parity and type of labour should be taken into account in risk adjustment models for interhospital comparison. Moreover, Robson’s classification proved to be useful to compare caesarean rates among hospitals even though the presence of residual confounding related to clinical and socio-demographic variables within strata may lead to potential bias, especially in low-risk nulliparous and multiparous women with spontaneous labour (groups I and III). Only after eliminating confounders in comparative evaluation of hospital performance we may be confident that we are considering unnecessary variability and inappropriate procedures. Unnecessary variability must be the target for health care quality improvement activities.

## Competing interest

The authors declare that they have no competing interests.

## Authors’ contributions

CP: study conception and design, data analysis, interpretation of data. FMP: study conception and design, interpretation of data. FD: study design, data analysis, interpretation of data. CE: data analysis. SE: data analysis. LJ: statistical analysis. PG: interpretation of data. PCA: study conception and design, interpretation of data. All authors read and approved the final manuscript.

## Pre-publication history

The pre-publication history for this paper can be accessed here:

http://www.biomedcentral.com/1471-2393/12/54/prepub

## Supplementary Material

Additional file 1 **Appendix A.** A) ICD 9-CM codes to identify clinical variables from mothers’ discharge records. B) ICD 9-CM codes to identify variables from neonatal discharge records. C) ) ICD 9-CM codes to identify variables from both neonatal and maternal discharge records. Click here for file
