# Supplementary material for: Risk adjustment models for interhospital comparison of CS rates using Robson’s ten group classification system and other socio-demographic and clinical variables
Source: BMC Pregnancy Childbirth. 2012 Jun 21;12:54. doi: 10.1186/1471-2393-12-54 (PMC3570355; doi:10.1186/1471-2393-12-54)
Supplement: Additional file 1 — Appendix A. A) ICD 9-CM codes to identify clinical variables from mothers’ discharge records. B) ICD 9-CM codes to identify variables from neonatal discharge records. C) ) ICD 9-CM codes to identify variables from both neonatal and maternal discharge records. [file 1471-2393-12-54-S1.doc]

**APPENDIX A**

*A) ICD 9-CM codes to identify clinical variables from mothers’ discharge records*

| **Description** | **ICD 9-CM code** |
| --- | --- |
| Severe co-morbid illness of the mother | 14-16, 170-176, 179, 18-19-20, 282.4, 282.6, 286, 287, 342, 344, 340, 3419, 39, 41, 42, 43, 441, 442, 446.4, 58, 646.21, 646.23, 648.5, 648.6, 659.3, 669.11, 669.13, 745-747 |
| HIV | 042, V08 |
| Diabetes | 250, 648.0, 648.8 |
| Hypertension | 405, 642.00, 642.01, 642.03, 642.1-642.2, 642.30, 642.31, 642.33, 642.90, 642.91, 642.93 |
| Lung disease | 01, 44.1, 44.2, 48-51, 647.30, 647.31, 647.33, 668.01 |
| Genital Herpes | 054.1 |
| Ante-partum haemorrhage/abruptio placentae/Placenta Praevia | 641 |
| Dystocia | 653, 660, 661 (except 661.3), 662 |
| Eclampsia/Pre-eclampsia | 642.40, 642.41, 642.43, 642.5, 642.60, 642.61, 642.71, 642.73 |
| Foetal-pelvic disproportion/excessive development of the infant | 653, 656.60, 656.61, 656.63 |
| Foetal anomalies | 655 |
| RH-Isoimmunisation | 656.1 |
| Polyhydramnios | 657 |
| Oligohydramnios | 658.0 |
| Premature rupture of the membranes | 658.1 |
| Other problems of the amnios | 658.4, 658.8, 658.9 |
| Abortion threads/assisted fecundation | 63, 640, 644.0, 646.3, V26, V23.0, V23.2, V23.4, V23.5, V23.7, V23.8 |

B) ICD 9-CM codes to identify variables from neonatal discharge records

| **Description** | **ICD 9-CM code** |
| --- | --- |
| Congenital Malformation | 740-759 |
| Post-maturity and Macrosomia | 766 |

C) ICD 9-CM codes to identify variables from both neonatal and maternal discharge records

| **Description** | **ICD 9-CM code** |
| --- | --- |
| Foetal distress | 656.3, 768 |
| Intrauterine growth retardation | 764, 656.5 |
